# Real-time multi-contrast magnetic particle imaging for the detection of gastrointestinal bleeding

**DOI:** 10.1038/s41598-023-50041-3

**Published:** 2023-12-27

**Authors:** Fabian Mohn, Patryk Szwargulski, Michael G. Kaul, Matthias Graeser, Tobias Mummert, Kannan M. Krishnan, Tobias Knopp, Gerhard Adam, Johannes Salamon, Christoph Riedel

**Affiliations:** 1 Section for Biomedical Imaging, University Medical Center Hamburg-Eppendorf, Hamburg, Germany; 2 Institute for Biomedical Imaging, Hamburg University of Technology, Hamburg, Germany; 3 Department of Diagnostic and Interventional Radiology and Nuclear Medicine, University Medical Center Hamburg-Eppendorf, Martinistrasse 52, 20246 Hamburg, Germany; 4 Fraunhofer Research Institution for Individualized and Cell-based Medical Engineering, IMTE, Lübeck, Germany; 5 Institute of Medical Engineering, University of Lübeck, Lübeck, Germany; 6 Department of Materials Science and Engineering, University of Washington, Seattle, USA

**Keywords:** Gastrointestinal bleeding, Gastrointestinal bleeding, Preclinical research

## Abstract

Gastrointestinal bleeding, as a potentially life-threatening condition, is typically diagnosed by radiation-based imaging modalities like computed tomography or more invasively catheter-based angiography. Endoscopy enables examination of the upper gastrointestinal tract and the colon but not of the entire small bowel. Magnetic Particle Imaging (MPI) enables non-invasive, volumetric imaging without ionizing radiation. The aim of this study was to evaluate the feasibility of detecting gastrointestinal bleeding by single- and multi-contrast MPI using human-sized organs. A 3D-printed small bowel phantom and porcine small bowel specimens were prepared with a defect within the bowel wall as the source of a bleeding. For multi-contrast MPI, the bowel lumen was filled with an intestinal tracer representing an orally administered tracer. MPI was performed to evaluate the fluid exchange between the vascular compartment of the bowel wall and the lumen while a blood pool tracer was applied. Leakage of the blood pool tracer was observed to the bowel lumen. Multi-contrast MPI enabled co-registration of both tracers at the same location within the bowel lumen indicating gastrointestinal bleeding. Single- and multi-contrast MPI are feasible to visualize gastrointestinal bleeding. Therefore, MPI might emerge as a useful tool for radiation-free detection of bleeding within the entire gastrointestinal tract.

## Introduction

Gastrointestinal (GI) bleeding represents as a clinical manifestation of a variety of diseases^1^. GI bleeding occurs within the entire GI tract and can be classified based on its anatomic location into upper, middle, and lower GI bleeding. Upper GI bleeding is located between mouth to duodenum above the ampulla of Vater^2^. Peptic ulcer disease followed by gastritis and esophagitis are the most common causes, while bleeding from esophageal varices and neoplasms are associated with the highest mortality^3^. A bleeding in the middle GI tract, between the ampulla of Vater to the terminal ileum^2^, might arise from vascular pathologies (e.g. angiodysplasia, Dieulafoy’s lesions), inflammatory bowel disease, Meckel’s diverticulum, ulcers or neoplasms^4–6^. A lower GI bleeding occurs in the colon or rectum^7^. The most common causes include colonic diverticulosis followed by internal hemorrhoids and ischemic colitis^8^.

A GI bleeding might present as an overt or as an occult bleeding. An overt bleeding is characterized by visible signs of a bleeding like hematemesis, hematochezia, or melena. Acute overt GI bleeding, as a potentially life-threatening condition, requires prompt clinical evaluation and treatment^1^. Typically, in both the upper and the lower GI tract endoscopy serves as initial modality (i.e. esophagogastroduodenoscopy and ano-/sigmoido-/colonoscopy) to locate the bleeding source with the advantage to enable therapeutic intervention^8,9^. However, if endoscopic evaluation is not feasible or inconclusive, further imaging techniques like computed tomography (CT) might be necessary^1,10^. The diagnostic evaluation of a bleeding in the middle GI tract depends on the clinical presentation of the patient. If an acute overt bleeding in the small bowel is suspected, ^99m^Tc-labeled red blood cell scintigraphy, CT angiography or, in case of a hemodynamically unstable patient, conventional catheter angiography might reveal the bleeding source by extravasation of a tracer or contrast agent^4^. An occult bleeding might present with iron deficiency anemia or a positive fecal occult blood test, but without visible signs of a bleeding^1,5,11^. Initially, colonoscopy and esophagogastroduodenoscopy might be performed to find a bleeding source in the lower or upper GI tract, respectively. If no bleeding source is detected, CT enterography, video capsule endoscopy (VCE) and double balloon endoscopy (DBE) can be used to evaluate the small bowel^1^. VCE enables the noninvasive evaluation of the entire small bowel. However, limitations of this technique include the inability to influence the GI passage and the possibility to miss clinically important lesions^4^. DBE is performed in sedation and enables deep intubation of the small bowel with the possibility for therapeutic intervention. Nonetheless, lesions might be missed if a total enteroscopy is not achieved^4^.

Here, a non-invasive, radiation-free imaging technique with coverage of the entire GI tract is desirable for bleeding detection. Magnetic Particle Imaging (MPI) is a technique for volumetric imaging at a high spatiotemporal resolution without ionizing radiation based on superparamagnetic iron oxide nanoparticles (SPIONs)^12,13^. Potential applications encompass e.g. the in vivo evaluation of blood flows^14,15^, the assessment of brain perfusion^16^, MPI-derived angiography^17^, and MPI-guided vascular interventions^18,19^. Furthermore, MPI enabled the detection of an induced acute GI bleeding in a murine model^20^. In comparison to other imaging modalities like CT, MPI offers the advantage of multi-contrast imaging^21^ with the possibility to visualize more than one tracer simultaneously or to exploit the physical link of the magnetization behavior to measure viscosity or temperature^22–24^. Multi-contrast MPI has been used to detect intracranial bleeding and to monitor the expansion of the hematoma in a murine model^22^.

In this study we demonstrate the feasibility of non-invasive MPI to visualize real-time tracer enhancement of a bowel wall and to detect gastrointestinal bleeding in human-sized organs. Therefore, single- and multi-contrast experiments were performed on 3D-printed phantoms in the scale of human small bowel and on ex vivo porcine small bowel specimens to visualize extravasation of a blood pool tracer to the bowel lumen. In multi-contrast experiments, an additional intraluminal tracer represented an orally administered tracer providing an anatomic reference of the affected bowel segment.

## Methods

To prove and validate the registration of GI bleeding with MPI, fundamentals on data acquisition and image reconstruction are explained first. Then, a verification of the used tracers is laid out to verify their separability for multi-contrast MPI with a dilution series. Afterwards, the phantom construction process and phantom experiments are described. Finally, ex vivo porcine small bowel specimen measurements are specified, and experiments are performed in analogy to the phantom experiments.

The following four experiments were conducted on both phantoms and ex vivo porcine small bowel specimen.

- Single-contrast (blood pool tracer only), bowel intact (control)
- Single-contrast (blood pool tracer only), bowel perforated
- Multi-contrast, bowel intact (control)
- Multi-contrast, bowel perforated.

The purpose of control measurements was to demonstrate the absence of a bleeding, so any registered bleeding in a perforated bowel experiment could be attributed to the actual perforation of the phantom or bowel specimen.

### Magnetic particle imaging

Visualization of the spatial distribution of SPIONs was performed on a pre-clinical MPI system (Bruker Biospin GmbH, Ettlingen, Germany) using a gradiometric *x*-receive coil with a bore diameter of 72 mm. The receive coil was connected to a custom developed receive chain that filters, amplifies, and samples the signal. The measurements were recorded with a gradient strength of *G* = diag(− 0.75, − 0.75, 1.5) T/m and drive field amplitudes *A*_x_ = *A*_y_ = *A*_z_ = 12 mT resulting in a field of view (FOV) of 32 × 32 × 16 mm^3^. The repetition time was 21.54 ms, resulting in the acquisition of 46 volumes per second. With the use of a custom online reconstruction software, the experiments were displayed in real-time to monitor the process and outcome of the measurements at the scanner^25^. The final image reconstructions were performed afterwards, where parameters were visually optimized to obtain the best results^26^.

### MPI image reconstruction

Multi-contrast reconstructions were performed using the iterative Kaczmarz method^27,28^, yielding the particle concentrations ${{\varvec{c}}}_{1}$ and ${{\varvec{c}}}_{2}$ by solving the linear system of equations

$$\widehat{{\varvec{S}}}{\varvec{c}}= \left[\begin{array}{cc}{\widehat{{\varvec{S}}}}_{1}& {\widehat{{\varvec{S}}}}_{2}\end{array}\right]\left[\begin{array}{c}{{\varvec{c}}}_{1}\\ {{\varvec{c}}}_{2}\end{array}\right]={\widehat{{\varvec{u}}}}_{{\text{receive}}},$$

where ${\widehat{{\varvec{u}}}}_{{\text{receive}}}\in {\mathbb{C}}^{K}$ is the Fourier transform of the received voltage with *K* frequency components and ${\widehat{{\varvec{S}}}}_{i}\in {\mathbb{C}}^{K\times N}$ for *i* = 1, 2 are the tracer specific system matrices in the frequency domain. The system matrices were acquired on a grid of 20 × 20 × 20 voxels (voxel size 2 × 2 × 1 mm^3^) in phantom experiments and on a grid of 26 × 26 × 26 voxels (voxel size 1.5 × 1.5 × 0.75 mm^3^) for the porcine small bowel specimens. This included an overscan of at least 2 voxels in all directions to reduce boundary artifacts^29^. In case of single-contrast reconstruction, only a single tracer is regarded and ${\widehat{{\varvec{S}}}}_{2}, {{\varvec{c}}}_{2}$ are neglected. For all reconstructions, the following least squares problem is solved

$${{\varvec{c}}}_{{\text{reco}}}^{\lambda }=\underset{{\varvec{c}}\epsilon {\mathbb{R}}_{+}^{N}}{{\text{argmin}}} {\Vert \widehat{{\varvec{S}}}{\varvec{c}}-{\widehat{{\varvec{u}}}}_{{\text{receive}}}\Vert }_{2}^{2}+\lambda {\Vert {\varvec{c}}\Vert }_{2}^{2},$$

where ${\Vert \widehat{{\varvec{S}}}{\varvec{c}}-{\widehat{{\varvec{u}}}}_{{\text{receive}}}\Vert }_{2}^{2}$ is the data discrepancy term and ${\Vert {\varvec{c}}\Vert }_{2}^{2}$ is the penalization term that is utilized to dampen large oscillations in the solution. The solution of the concentration distribution ${{\varvec{c}}}_{{\text{reco}}}^{\lambda }$ contains both concentration vectors ${{\varvec{c}}}_{1}$ and ${{\varvec{c}}}_{2}$ and is used to display the distributions in different colors. Therefore, voxels that contain signal from both tracers appear as a blend of these two colors. $\lambda \epsilon {\mathbb{R}}_{+}$ controls the relative regularization parameter that reduces the image noise at the cost of spatial resolution. The frequency selection, the parameter λ, and the number of iterations were adapted individually for each reconstruction to obtain best image results. However, parameters were identical for both tracers as the two system matrices constituted to a single minimization problem. For visualization purposes, images were averaged to a temporal resolution of 1.72 s (80 averages) to reduce noise.

### Post-processing of MPI reconstructions

Post-processing techniques might be used to improve the visualization of tracer extravasation and consequently increase the sensitivity for bleeding detection. For single-contrast MPI, digital subtraction images were calculated by voxel-wise subtraction of signal intensities at two different timepoints (*t*_2 _− *t*_1_). Consequently, only the temporal changes in signal intensities between both timepoints were visible. For multi-contrast MPI, overlay images highlighting a mixture of both tracers were reconstructed. Therefore, only voxels with signal intensities of both tracers above a defined threshold were displayed. The thresholds were visually determined to obtain the best delineation of the tracer mixture. Here, MPI at a single timepoint is sufficient to calculate this image.

### Tracer separability for multi-contrast MPI

In this study, the tracer Perimag® (Micromod Partikeltechnologie GmbH, Rostock, Germany) was used as blood pool tracer and LS-008 (Lodespin Labs, Seattle, United States) was used as intestinal tracer. LS-008 is a single-core tracer with polyethylene glycol coating^30,31^, whereas Perimag is a multi-core tracer with dextran coating^32^. To separate their signal contributions to the receive signal ${\widehat{{\varvec{u}}}}_{{\text{receive}}}$, an image of a specimen was reconstructed using both system matrices to plot each tracer’s intensity. Before actual experiments were performed, a dilution series was used to compare the signal contribution in known samples of different tracer compositions. Specimens of 100 µl were prepared at different volume ratios of both tracers, Perimag (at *c*_Fe_ = 266 µg/ml (4.75 mmol/l)) and LS-008 (at *c*_Fe_ = 321 µg/ml (5.75 mmol/l)). Concentrations were chosen to obtain similar peak signal intensities and to match the signal-to-noise ratio of both tracers which prevents signal dominance of one tracer over the other. Volume ratios of Perimag/LS-008 were set in 10% steps (100/0, 90/10, 80/20, …, 10/90, 0/100) to evaluate the channel leakage and tracer separability by their signal contributions after image reconstruction. Due to the underlying linear signal model of MPI^13^, the signal intensity of each channel is expected to scale linearly from 0 to 100%.

### 3D-printed small bowel phantom experiments

**Figure 1:**
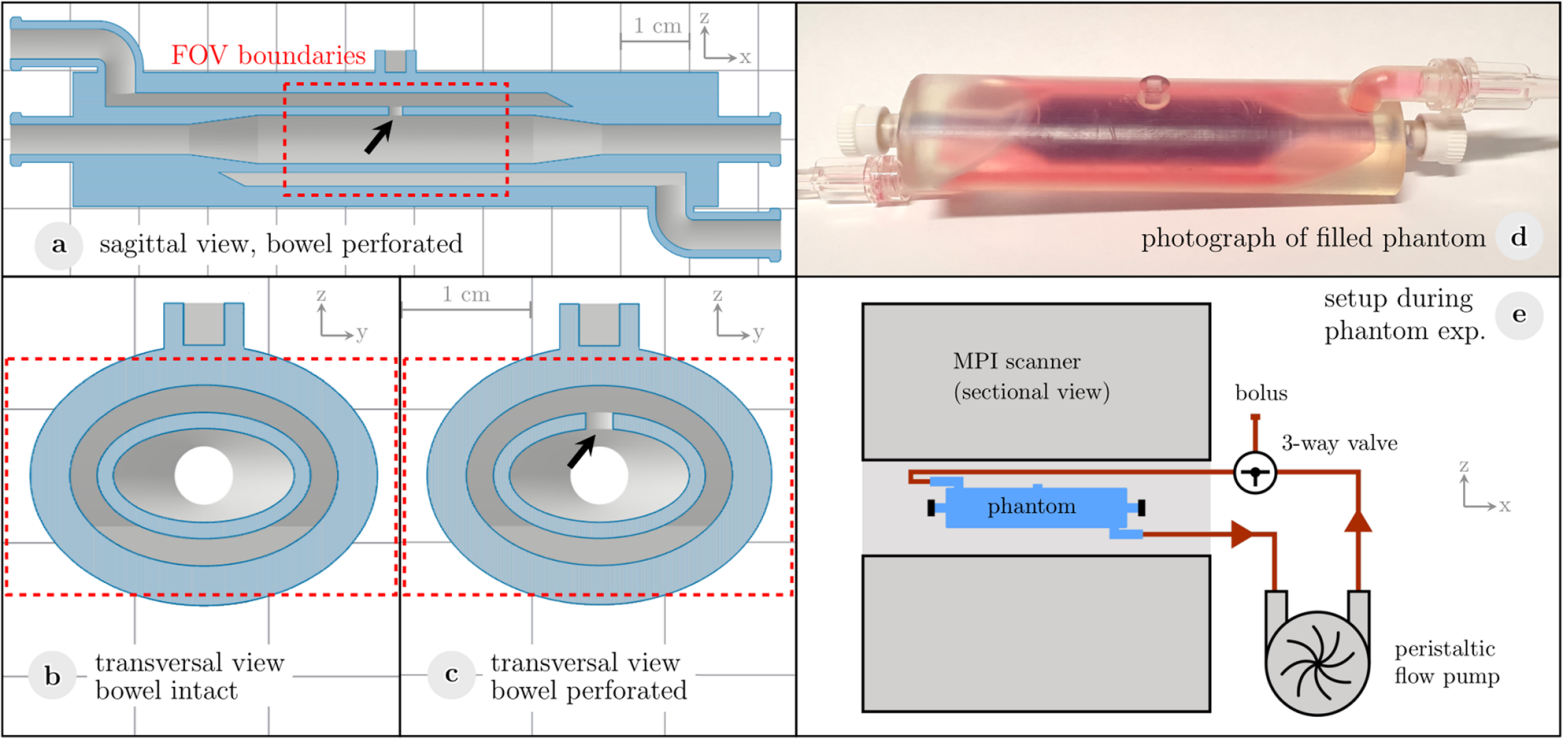
Phantoms of a small bowel segment. (**a**) Sectional view of the phantom illustrates the design of the small bowel phantom in a sagittal cross section consisting of an inner bowel lumen and an outer vascular compartment. The vascular compartment was accessible via an inlet and an outlet to enable the connection to a circulatory system. (**b**) The transversal cross section of the control phantom shows a separation of the vascular and the luminal compartment. (**c**) Another phantom was created with a perforation (arrow) between both compartments representing the source of a bleeding. (**d**) Photograph of a phantom with separated compartments, dyed for illustration (blue = bowel lumen, red = vascular compartment). (**e**) The phantoms were placed within the MPI scanner and the vascular compartment was connected to a circulatory system.

Phantoms resembling a small bowel segment were 3D-printed using clear resin in a stereolithography printer (Form 3, Formlabs Inc., Somerville, USA). Coated with NanoSeal (NanoSeal LLC, Conroe, USA), the printed structures were hydrophobic and impermeable for liquids. The core structure of the phantom had the shape of an oval cylinder (Fig. 1a–d). The interior oval lumen measured 7.0 mm × 13.5 mm which is in the range of human small bowel^33^. The surrounding bowel wall was constructed with a hollow layer of 2.0 mm representing the vascular compartment in a perfused bowel wall. One phantom was created with a separation of the vascular and the luminal compartment and served as control (Phantom I, Fig. 1b). Another phantom was created with a perforation of 2.0 mm between both compartments representing the source of a bleeding (Phantom II, Fig. 1c). The luminal compartment was filled with water for single-contrast MPI and with a tracer suspension of LS-008 (*c*_Fe_ = 321 µg/ml (5.75 mmol/l)) as intestinal tracer for multi-contrast MPI. During the experiments, the inlet and outlet of the lumen were sealed. The vascular compartment was accessible via an inlet and an outlet. Both inlet and outlet were connected to a water-filled circulatory system (total volume 15 ml) with a flow pump and a three-way valve enabling the injection of a blood pool tracer (Fig. 1e). The peristaltic flow pump was set to a flow rate of 93 ml/min. The phantoms were placed at the center of the MPI scanner by guidance of a small delta sample mounted on the phantoms. Phantoms were imaged dynamically in the MPI scanner to evaluate the exchange process between the vascular compartment and the lumen while a bolus of 1 ml Perimag (*c*_Fe_ = 850 µg/ml (15.2 mmol/l)) was injected as the blood pool tracer.

The following experiments were conducted:

- Single-contrast (blood pool tracer only), bowel intact (phantom I)
- Single-contrast (blood pool tracer only), perforated bowel (phantom II)
- Multi-contrast, bowel intact (phantom I)
- Multi-contrast, perforated bowel (phantom II)

### Porcine small bowel specimens and ex vivo experiments

**Figure 2:**
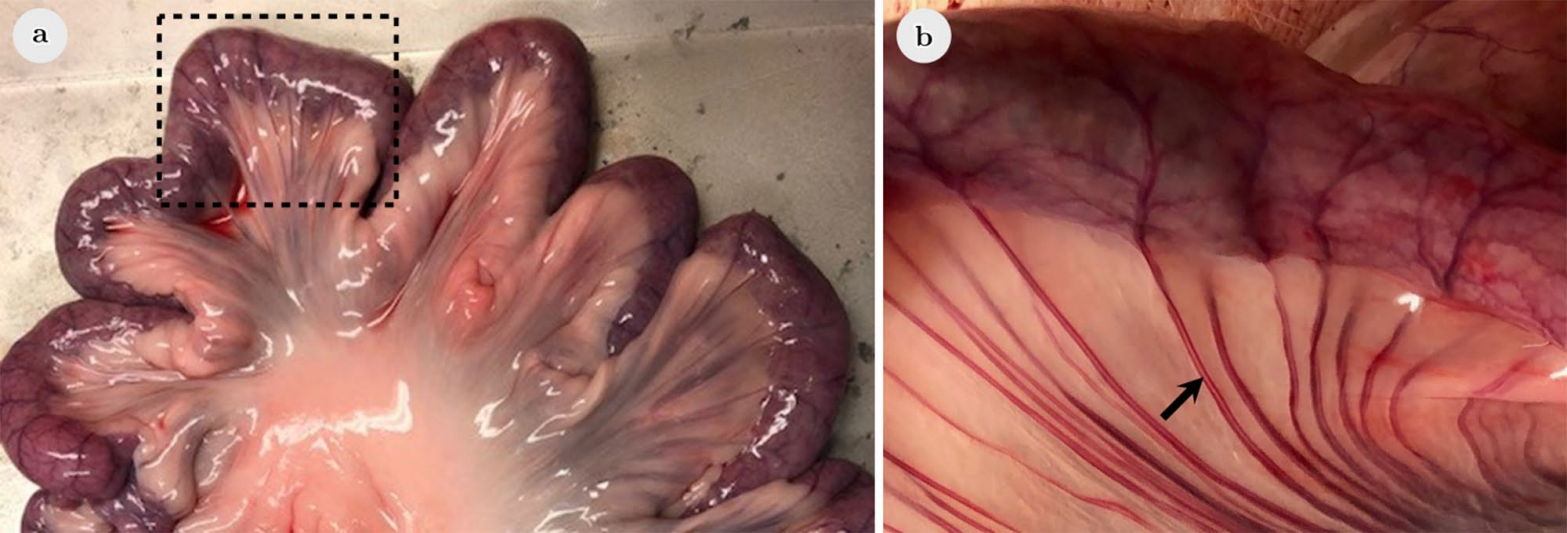
Photograph of porcine ex vivo small bowel specimen. (**a**) Porcine small bowel was used for preparation of small bowel specimen of 3–4 cm (rectangle). (**b**) An adjacent mesenteric vessel was cannulated to enable injection of the blood pool tracer (arrow).

In analogy to the four phantom experiments, similar ex vivo experiments for single- and multi-contrast MPI were performed using porcine small bowel specimens (Fig. 2a) of a female pig (German landrace, approx. 85 kg). The pig was euthanized at the University Medical Center Hamburg-Eppendorf within the scope of a different study approved by the national authority for animal protection (Behörde für Gesundheit und Verbraucherschutz, Hamburg, Germany). It conforms to the Guide for the Care and Use of Laboratory Animals, eighth edition, updated by the US National Research Council Committee in 2011 and was performed in accordance with the European Directive 2010/63 EU. The original approval does not address our experiments. Subsequent experiments on residual cadaver tissue are allowed without additional approval in Germany. To avoid further animal experiments, the residual cadaver tissue of the pig was used to prepare small bowel specimens. No animal was euthanized within the scope of this study. Small bowel specimens were prepared of a length of 3–4 cm including the adjacent mesenteric tissue. An incision in the mucous membrane of a length of 0.5 cm represented the source of a bleeding. Specimens with an intact mucous membrane served as control. Small bowel specimens were ligated at both ends and a mesenteric vessel was cannulated using a 24G catheter to enable the injection of a blood pool tracer (Fig. 2b). The bowel lumen was filled with water for single-contrast MPI and with a tracer suspension of LS-008 as intestinal tracer for multi-contrast MPI. The specimens were placed at the center of the MPI scanner and imaged dynamically in the MPI scanner to evaluate the exchange process between the vascular compartment and the lumen while a bolus of 1 ml Perimag was injected manually through the cannulated mesenteric vessel followed by slow injection of 1 ml water.

## Results

### Tracer separability

**Figure 3:**
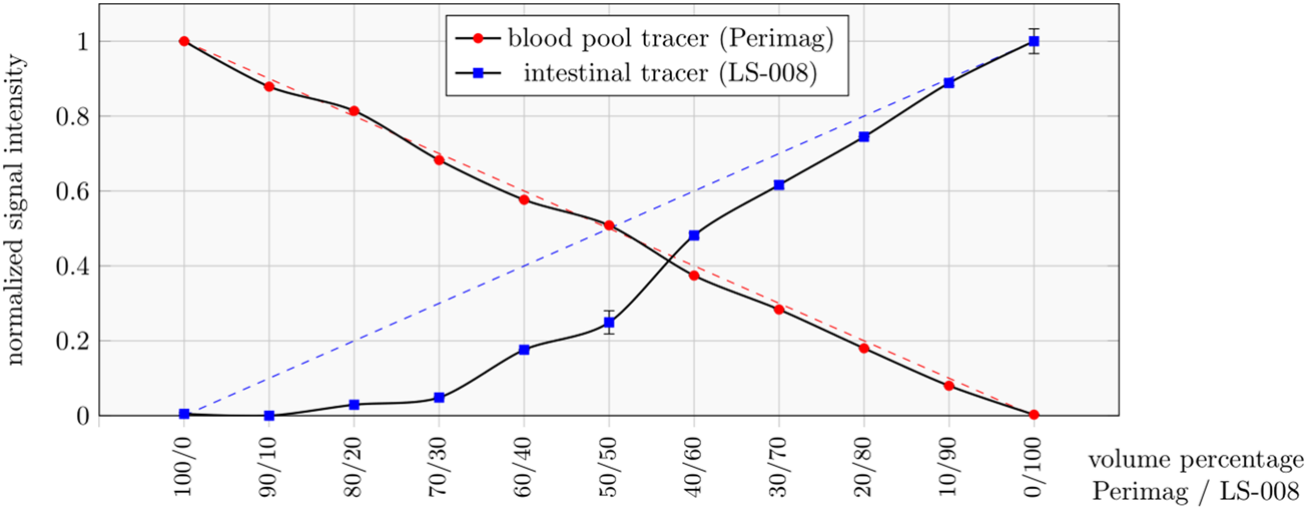
Multi-contrast MPI-derived signal intensities of different volume ratios of Perimag/LS-008 in equidistant 10% steps-contrast. MPI-derived signal intensity mean and variance of different volume ratios of Perimag/LS-008 in equidistant 10% steps obtained from 20 measurements (frames). Both curves are normalized with their maximum signal intensity and dashed lines indicate the expectation based on the linear MPI model.

Image reconstructions using the MPI multi-contrast approach of 100 µl samples provided the signal intensities of different volume ratios of Perimag/LS-008 in equidistant 10% steps shown in Fig. 3. The multi-contrast reconstruction of 100% Perimag resulted only in a signal in the Perimag channel without relevant signal leakage to the LS-008 channel. A decreasing volume fraction of Perimag led to a strictly monotonically decreasing signal intensity of Perimag. At a volume ratio of 90% Perimag/10% LS-008, the LS-008 signal was not detectable. At higher concentrations of LS-008, an increasing volume fraction of LS-008 led to a strictly monotonically increasing signal intensity of LS-008. The multi-contrast reconstruction of 100% LS-008 resulted only in a signal in the LS-008 channel without signal leakage to the Perimag channel. A mixture of both tracers could be clearly determined at volume ratios between 80% Perimag/20% LS-008 and 10% Perimag/90% LS-008.

### Phantom experiments

**Figure 4:**
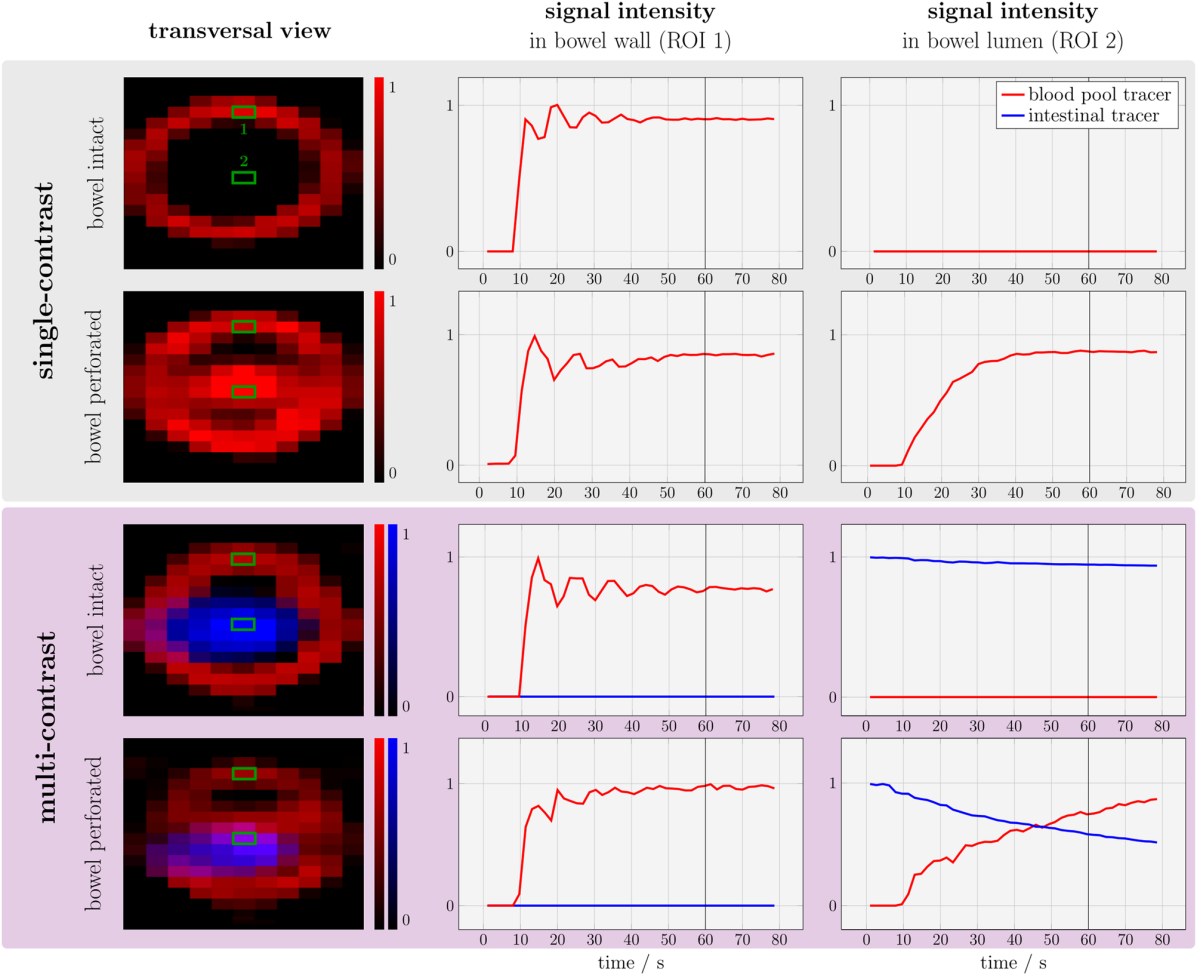
MPI of small bowel phantoms. Images on the left show MPI reconstructions at 60 s. MPI enabled visualization of the enhancing bowel wall after injection of the blood pool tracer (red). In case of multi-contrast MPI, the luminal compartment is visualized by the intestinal tracer (blue). A representative region of interest (ROI) in the bowel wall (ROI 1) and the lumen (ROI 2) was identified manually. A signal increase of the blood pool tracer within the bowel lumen was only observed in case of a perforation between the vascular and the luminal compartment and indicated GI bleeding. For multi-contrast MPI, leakage of the blood pool tracer led to co-registration of both tracers at the same location within the lumen and indicated GI bleeding.

MPI enabled real-time 3D tracer visualization in all phantom experiments. The measured signal intensities of phantom experiments for the four different scenarios, intact bowel vs. perforated bowel using both single- and multi-contrast MPI, are shown in Fig. 4. A side-by-side comparison of single- and multi-contrast MPI of small bowel phantoms with an intact and a perforated bowel wall is shown in Supplementary Video S1. After injection at 10 s, the signal of the blood pool tracer increased within the bowel wall and reached a plateau phase in all experiments.

Single-contrast measurements with an intact bowel wall did not detect any tracer within the bowel lumen. Single-contrast measurements with a perforated bowel wall revealed a signal increase of the blood pool tracer within the bowel lumen after injection caused by extravasation of the tracer from the vascular to the luminal compartment. The extravasation was observed immediately after bowel wall enhancement. Within the lumen, the signal intensity was higher at the bottom of the lumen due to sedimentation of the tracer.

Multi-contrast MPI enabled the visualization of the bowel lumen before injection of the blood pool tracer. After tracer injection to the intact bowel phantom, the signal of the intestinal tracer remained nearly constant without any detectable blood pool tracer within the bowel lumen. Multi-contrast MPI of the perforated bowel phantom revealed a co-registration of the signal of both vascular and intestinal tracer at the same location within the lumen. This leakage of the blood pool tracer with consecutive mixture of both tracers indicated GI bleeding. The mixture was observed immediately after bowel wall enhancement. The signal intensity of the intestinal tracer decreased overtime, being partially diluted by the inflowing blood pool tracer through the perforation.

**Figure 5:**
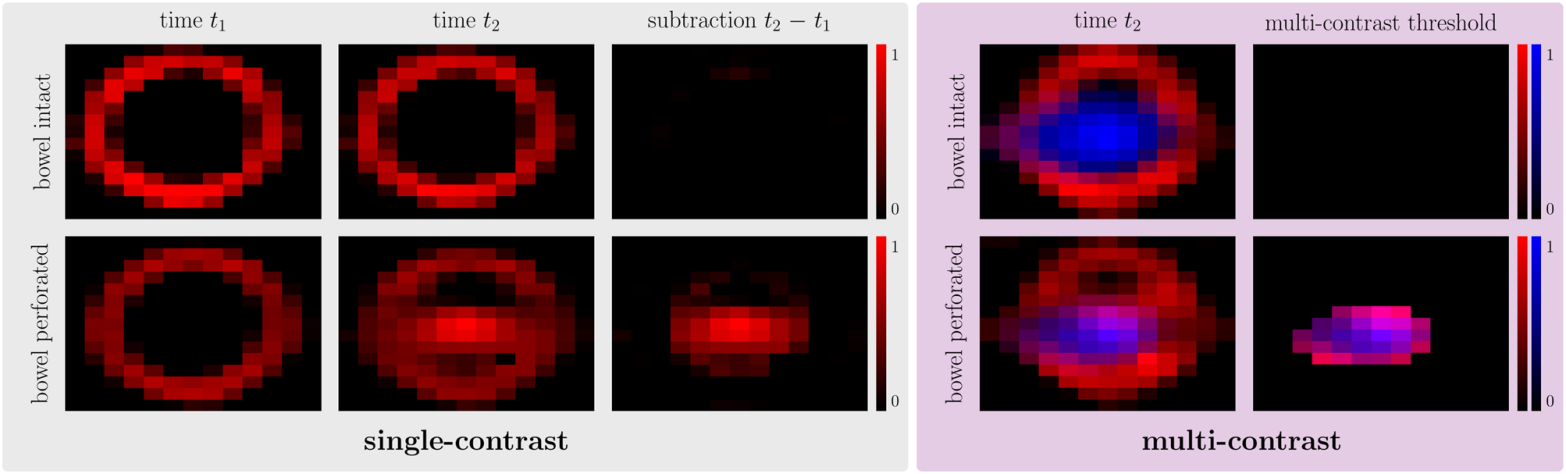
Post-processed single- and multi-contrast MPI of small bowel phantoms. Left: Digital subtraction of the reconstructed images at t_2_ = 60 s and immediately after bowel wall enhancement at t_1_ = 15 s. A GI bleeding was not detected for the intact bowel wall. The perforated bowel wall led to a residual signal after digital subtraction indicating GI bleeding. Right: Overlay of multi-contrast MPI-derived signal intensities above a defined threshold. The thresholds were visually determined to obtain the best delineation of the tracer mixture with the used setup and reconstruction parameters (threshold blood pool tracer: 28% of the maximum signal intensity; threshold intestinal tracer: 38% of the maximum signal intensity). Co-registration of both tracers at the same location was only observed in case of a perforated bowel wall and indicated GI bleeding.

Digital post-processing enabled a clear identification of GI bleeding in single- and multi-contrast MPI of small bowel phantoms (Fig. 5). Digital subtraction of reconstructed single-contrast images at *t*_2_ = 60 s and immediately after bowel wall enhancement at *t*_1_ = 15 s does not show a GI bleeding for the intact bowel wall. The perforated bowel wall led to a residual signal after digital subtraction indicating GI bleeding. For multi-contrast MPI, overlay of multi-contrast MPI-derived signal intensities above a defined threshold of both the intestinal tracer and the blood pool tracer clearly indicated GI bleeding only in case of a perforated bowel wall.

### Small bowel ex vivo experiments

**Figure 6:**
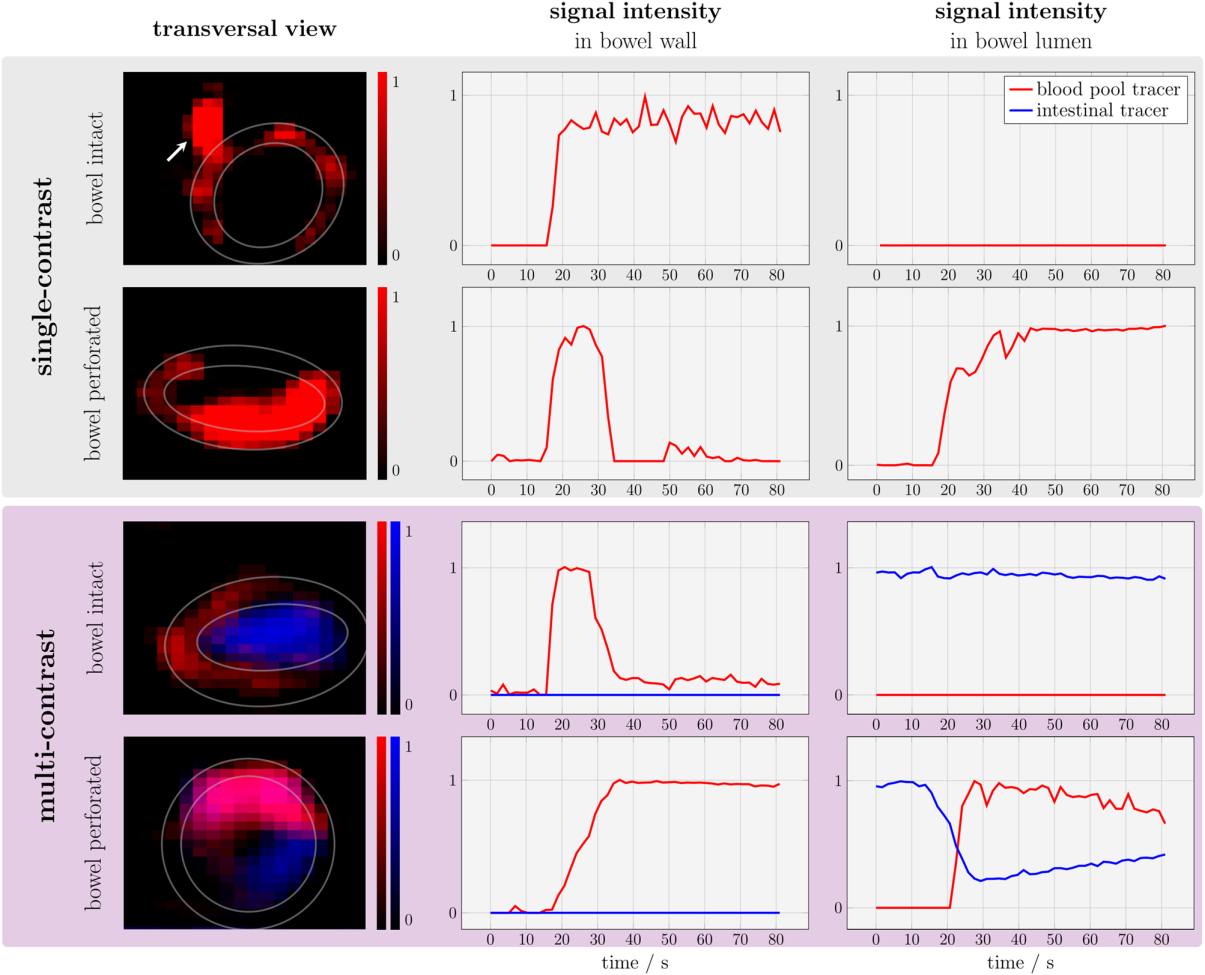
MPI of ex vivo porcine small bowel specimen. These experiments were conducted in analogy to phantom experiments. Single contrast MPI enabled visualization of the enhancing bowel wall after bolus injection. Note the enhancement of the feeding vessel for this bowel segment (arrow). A signal increase of the blood pool tracer within the bowel lumen was only detected after incision in the mucous membrane. In multi-contrast MPI, co-registration of both tracers at the same location in the lumen represented the mixture of tracers and indicated GI bleeding.

Single- and multi-contrast MPI enabled real-time 3D tracer visualization in ex vivo porcine small bowel specimens during tracer injection. The signal intensities of ex vivo small bowel measurements for the four different scenarios, intact bowel vs. perforated bowel using both single- and multi-contrast MPI, are shown in Fig. 6. After injection of the blood pool tracer, the signal increased partially within the bowel wall, but without enhancement of the entire circumference.

Single-contrast measurements with an intact mucous membrane did not detect any tracer within the bowel lumen. Single-contrast measurements after incision of the mucous membrane revealed a signal increase of the blood pool tracer within the bowel lumen after injection caused by extravasation of the tracer from the vascular to the luminal compartment. The extravasation was observed immediately after bowel wall enhancement. Similar to the corresponding phantom experiment, the signal intensity was higher at the bottom of the lumen due to sedimentation of the tracer.

Using a blood pool tracer and an intestinal tracer, multi-contrast MPI enabled the visualization of the bowel wall and the lumen. After injection of the blood pool tracer to the intact bowel segment, the signal increased partially within the bowel wall, but without enhancement of the entire circumference. The signal of the intestinal tracer remained nearly constant without a detectable signal increase of the blood pool tracer within the bowel lumen. Multi-contrast MPI after incision of the mucous membrane showed an extravasation of the blood pool tracer to the lumen with partial displacement of the intestinal tracer and mixture of both tracers. The mixture led to a co-registration of the signal of both blood pool tracer and intestinal tracer at the same location within the lumen. This leakage of the blood pool tracer with consecutive mixture of both tracers indicated GI bleeding. The mixture was observed immediately after bowel wall enhancement.

The subsequent injection of water led to a signal decrease of the bowel wall in two experiments (i.e., single-contrast MPI with incision of the mucous membrane and multi-contrast MPI with an intact bowel wall), indicating a washout of the blood pool tracer. For single-contrast MPI with an intact bowel wall and multi-contrast MPI with incision of the mucous membrane, parts of the blood pool tracer remained within the bowel wall.

## Discussion

We successfully demonstrated the feasibility of MPI to detect gastrointestinal bleeding in a human-sized bowel in real-time. MPI enabled visualization of the tracer enhancing bowel wall in both 3D-printed bowel phantoms and ex vivo porcine bowel specimen. Accumulation of the blood pool tracer within the bowel lumen as an indicator for GI bleeding was observed using single-contrast imaging. Multi-contrast MPI enabled detection of GI bleeding by co-registration of both tracers at the same location within the bowel lumen.

GI bleeding, as a potentially life-threatening condition, requires prompt clinical evaluation^1^. Therefore, a noninvasive technique for comprehensive real-time imaging of the entire GI tract is desirable. Esophagogastroduodenoscopy or colonoscopy might serve as initial modality to locate the bleeding source^8,9^, but do not enable assessment of the entire GI tract. Further, endoscopic techniques might require bowel preparation and patient sedation^1^. Other techniques like CT and catheter angiography enable bleeding detection in the entire GI tract but require the use of ionizing radiation. MPI does not apply ionizing radiation and is therefore potentially safe for patients if applied magnetic fields stay within safety limits^12^.

Similar to CT, tracer extravasation to the bowel lumen indicates GI bleeding. In our study, the detection of tracer extravasation was already possible after image reconstruction. In more complex cases, such as small GI bleedings, the additional use of post-processing techniques might enable a superior visualization of GI bleedings and consequently increase the sensitivity for bleeding detection. However, future studies need to prove a beneficial detection rate before implementation of these post-processing techniques.

Unfortunately, if an intravascular MPI tracer is cleared from the blood pool, single-contrast MPI does not provide an anatomical reference and allocation of tracer extravasation to a specific bowel segment is not possible. Here, the use of a long-circulating blood pool tracer might help to overcome this issue^34–37^. After reaching a steady state blood pool concentration of the tracer, a first MPI scan could provide the anatomical reference. A repeated MPI scan with subsequent image subtraction might enable visualization of tracer accumulation within the bowel lumen. Multi-contrast MPI with the use of a second, intestinal tracer is an alternative method to obtain an anatomical reference. After oral administration, MPI enables the detection of tracer-filled bowel. The mixture of both intestinal and blood pool tracer within the bowel lumen indicates GI bleeding. Here, GI bleeding detection might also be improved with a long-circulating blood pool tracer. In case of slow bleeding, MPI might be performed or repeated after a defined period of time when the amount of tracer extravasation is considered to be detectable. In case of an intermittent bleeding, MPI could be performed or repeated when bleeding is suspected to be active. Measurements can be repeated multiple times without the need of ionizing radiation. Thus, the sensitivity of GI bleeding detection might be improved with the multi-contrast approach.

All experiments were performed on a pre-clinical MPI system. This system enabled the investigation of a FOV encompassing small bowel phantoms in a human-sized scale or porcine small bowel segments. However, the basic principle of bleeding detection by extravasation of a blood pool tracer to the lumen applies to the entire GI tract. Therefore, MPI would enable bleeding detection within the entire GI tract including esophagus, stomach, colon and rectum if adequate MPI systems with a FOV encompassing the entire abdomen are developed for clinical applications^38^.

With regard to limitations, motion-related artifacts (e.g. caused by bowel or patient movements) were not considered, since all experiments were performed on phantoms or ex vivo bowel specimen. These artifacts cause blurring in averaged images and consecutive reduction of image quality but might not have a major influence due to the high temporal resolution of MPI. However, bleeding detection by post-processing techniques using images of more than one timepoint (i.e. digital subtraction) will be impaired due to bowel or patient movement. Here, multi-contrast MPI has the advantage to enable co-registration of a blood pool tracer and an intraluminal tracer within the bowel lumen at a single timepoint and has a higher robustness against such motion-related artifacts.

Tracer separability was confirmed by a dilution series to demonstrate that multi-contrast MPI enabled co-registration of two tracers in the same voxel. This feature was used to detect extravasation of the blood pool tracer to the lumen with consecutive mixture with the intestinal tracer. Depending on the voxel size, partial volume effects at the border of the lumen to the bowel wall might occur and imitate a mixture of both tracers. Such partial volume effects have not been investigated in this feasibility study.

Experiments were performed on porcine ex vivo small bowel specimen. After injection of the blood pool tracer, only parts of the bowel wall enhanced. A possible explanation for this phenomenon might be an occlusion of capillaries during specimen preparation. Here, future in vivo studies will potentially observe a more uniform bowel wall enhancement.

In conclusion, MPI enables the visualization of tracer enhancement of the bowel wall in bowel phantoms and ex vivo porcine bowel specimen. Further, both single- and multi-contrast MPI enable the detection of a GI bleeding. Therefore, MPI might emerge as a useful tool for noninvasive, radiation-free detection of acute and chronic GI bleeding if adequate MPI systems for clinical use are developed and suitable MPI tracers are approved.

### Supplementary Information

Supplementary Video S1.

## Data Availability

The datasets generated during and/or analyzed during the current study are available from the corresponding author on reasonable request.
